# A QSAR–ICE–SSD Model Prediction of the PNECs for Per- and Polyfluoroalkyl Substances and Their Ecological Risks in an Area of Electroplating Factories

**DOI:** 10.3390/molecules26216574

**Published:** 2021-10-30

**Authors:** Jiawei Zhang, Mengtao Zhang, Huanyu Tao, Guanjing Qi, Wei Guo, Hui Ge, Jianghong Shi

**Affiliations:** 1 State Environmental Protection Key Laboratory of Integrated Surface Water-Groundwater Pollution Control, School of Environmental Science and Engineering, Southern University of Science and Technology, Shenzhen 518055, China; 11750007@mail.sustech.edu.cn (J.Z.); 11652003@mail.sustech.edu.cn (M.Z.); 11850006@mail.sustech.edu.cn (H.T.); 11849066@mail.sustech.edu.cn (G.Q.); gwfybj@bjut.edu.cn (W.G.); 2 Environmental Engineering Research Centre, Department of Civil Engineering, The University of Hong Kong, Hong Kong 999077, China; 3 Key Laboratory of Beijing for Water Quality Science and Water Environment Recovery Engineering, Beijing University of Technology, Beijing 100124, China

**Keywords:** PFASs, QSAR–ICE–SSD, electroplating industry, ecological risk assessment

## Abstract

Per- and polyfluoroalkyl substances (PFASs) are a class of highly fluorinated aliphatic compounds that are persistent and bioaccumulate, posing a potential threat to the aquatic environment. The electroplating industry is considered to be an important source of PFASs. Due to emerging PFASs and many alternatives, the acute toxicity data for PFASs and their alternatives are relatively limited. In this study, a QSAR–ICE–SSD composite model was constructed by combining quantitative structure-activity relationship (QSAR), interspecies correlation estimation (ICE), and species sensitivity distribution (SSD) models in order to obtain the predicted no-effect concentrations (PNECs) of selected PFASs. The PNECs for the selected PFASs ranged from 0.254 to 6.27 mg/L. The ΣPFAS concentrations ranged from 177 to 983 ng/L in a river close to an electroplating industry in Shenzhen. The ecological risks associated with PFASs in the river were below 2.97 × 10^−4^.

## 1. Introduction

Per- and polyfluoroalkyl substances (PFASs) consist of carbon chains of different lengths where the hydrogen atoms are completely (perfluorinated) or partly (polyfluorinated) substituted by fluorine atoms. PFASs are widely used in the textile/leather treatment industry, manufacture of fluoropolymers, semiconductor industry, and electroplating industry. From 1951 to 2015, an estimated 2610–21,400 t of long-chain perfluoroalkyl carboxylic acids (PFCAs) were produced [1]. Due to the toxic effects, tissue accumulation, long-range transport, and environmental persistence of PFASs, perfluorooctanesulfonic acid (PFOS) was listed under the Stockholm Convention on Persistent Organic Chemicals and perfluorooctanoic acid (PFOA) was being considered for listing by 2017 [2]. As a result, around 3 million companies developed PFAS alternatives, for which they claim intellectual property rights protection [2].

An ecological risk assessment (ERA) aims to qualitatively or quantitatively describe the possibility that adverse ecological effects occur because of exposure to one or more stressors (e.g., chemical substances) [3]. An ERA has been adopted as an important methodology in many studies of typical PFASs such as PFOS [4] and PFOA [5]. The predicted no-effect concentration (PNEC) is expressed as the lowest concentration of adverse effects in an ecosystem of a given chemical substance [6]. The ratio of the PNEC to the measured exposure concentration (MEC) is known as the risk quotient (RQ), which is a screening-level descriptor of the ecological risk. To reduce the uncertainty associated with an ERA, the species sensitivity distribution (SSD) method is widely used to derive the PNEC [7,8]. An SSD is a cumulative probability distribution of the toxicity measurements of a chemical obtained from single-species bioassays of various species that can be used to estimate the ecotoxicological impacts of a chemical [9]. The robustness and accuracy of the SSD method strongly depend on the amount of species toxicity data [6,10,11]. Owing to the wide variety of PFASs and many emerging alternatives, the acute toxicity data for emerging PFASs and their alternatives are relatively limited [12]. The combination of quantitative structure-activity relationship (QSAR) models with interspecies correlation estimation (ICE) models can greatly expand the ability to predict untested chemicals and their potential effects on untested species; this has aroused a wide research interest [13,14,15,16]. QSAR models provide opportunities to estimate the ecotoxicity values for certain species (usually standard test species such as zebrafish) based on the knowledge of chemical structures or properties [17]. ICE models use available toxicity data of tested species (i.e., surrogate species) to predict those of untested species (i.e., predicted species) [18]. QSAR–ICE models can fill the data gap to generate SSDs, providing practical applications for the ERA of chemicals with limited data [13].

In aquatic systems, PFAS concentrations are higher in industrialized and urbanized areas than in less populated and remote regions in China [19]. Our previous study investigated the concentrations of PFOA and PFOS in the effluent of a sewage treatment plant in Beijing, which were found to be 29.9–71.5 ng/L and 60.1–233 ng/L, respectively [20]. These results indicated that the activated sludge process could not effectively remove PFOA and PFOS. Another previous study in the Fenhe River in Shanxi Province showed that the PFOA and PFOS concentrations were 2.49–4.79 ng/L and 3.54–16.2 ng/L, respectively [21]. Yamazaki et al. [22] reported that the PFAS concentrations ranged from non-detected to 1.5 ng/L in rivers and lakes on the Qinghai–Tibet Plateau, corresponding with low industrial levels. Based on the estimations of Wang et al. [19], the electroplating industry was the most important source of PFASs discharged into the aquatic environment.

Few studies have been undertaken on the occurrence and ERA of PFASs in the surface waters surrounding areas where electroplating industries operate. Relatively limited toxicity data make it difficult to develop the ERA of PFASs and their alternatives. In addition, QSAR–ICE–SSD models developed for estimating the PNECs of PFASs and their alternatives have been rarely reported in recent studies. Accordingly, the objectives of this study are (1) to construct QSAR–ICE–SSD models to predict the PNECs of PFASs and their alternatives and (2) to assess the ecological risk of PFASs in a river near electroplating factories.

## 2. Materials and Methods

### 2.1. Construction of QSAR–ICE Models

Following the procedures recommended in the Technical Guidance Document on Risk Assessment of the European Commission [6], the Guidelines for Ecological Risk Assessment of United States Environmental Protection Agency (US EPA) [3], and the literature [23,24,25], the process of collecting toxicity data can be summarized briefly as follows: four species (*Pseudokirchneriella subcapitata*, *Chlorella vulgaris*, *Daphnia magna*, and *Danio rerio*) representing three trophic levels in the aquatic environment were selected as model species for QSAR models. The acute toxicity data were mainly obtained from the US EPA ECOTOX database (http://cfpub.epa.gov/ecotox/ (accessed on 4 May 2021)), the literature, and relevant government documents. Structurally similar chemicals in the same group (i.e., PFASs) were used in the QSAR modeling. Chemicals that contained at least one -CF_2_- were originally considered as PFASs and further checked against the list of PFASs of the US EPA (https://comptox.epa.gov/dashboard/chemical_lists/pfasmaster (accessed on 4 May 2021)) [26]. Data screening followed the principles of accuracy, relevance, and reliability [27]. The test methods were in accordance with standard test methods (e.g., the methods of the Organization for Economic Cooperation and Development). The toxicity endpoints were the median lethal concentration (LC_50_) or the median effect concentration (EC_50_). The 48 h LC(EC)_50_ was preferred for invertebrate species and the 96 h LC(EC)_50_ was preferred for other species. When multiple toxicity values were available for the same species and the same endpoint, the geometric mean was taken as the mean toxicity value for the species.

Molecular structure files were obtained from the ChemSpider database (https://chemspider.com/ (accessed on 10 May 2021)) and the molecular energy was optimized using the GAMESS Interface method in ChemBio3D (https://perkinelmerinformatics.com/ (accessed on 12 May 2021)). A total of 12 semi-empirical molecular descriptors were then calculated using the AM1 method in MOPAC 2016 (http://openmopac.net/ (accessed on 12 May 2021)) and the *K*_ow_ values (shown in Table 1) were calculated using EPI Suite software (https://www.epa.gov/ (accessed on 12 May 2021)). The Chemical Abstracts Service Registry Number (CAS No.), chain lengths, and molecular descriptor values of the selected PFASs are listed in the Supplementary Excel file. There were 27 PFASs selected, in which chain lengths ranged from 2 to 15 and contained PFCAs, perfluoroalkane sulfonic acids (PFSAs), polyfluoroalkyl ether sulfonic acids (PFESAs), cyclic perfluorinated acids, fluorotelomer-based substances, and perfluoroalkyl acid precursors.

The stepwise regression method in SPSS (https://www.ibm.com/ (accessed on 4 May 2021)) was used to establish the multiple regression statistical models between the logarithmic values of the toxicity data (i.e., log LC(EC)_50_) and the molecular descriptors (including their logarithmic values)). Four QSAR models were validated using SIMCA software (https://www.sartorius.com/ (accessed on 28 May 2021)), in which the non-cross-validation correlation coefficient (r^2^) and leave-one-out cross-validation correlation coefficient (q^2^) were used as the evaluation indices.

A total of 227 ICE models of native species in China were established and used to estimate the acute toxicity data of 6 chemicals, including 4-dichlorophenol, triclosan, tetrabromobisphenol A, nitrobenzene, PFOS, and octachlorodiphenyl [28]. These ICE models were used after verifying the application domain.

### 2.2. Sample Treatment and Analysis of PFASs

**Figure 1:**
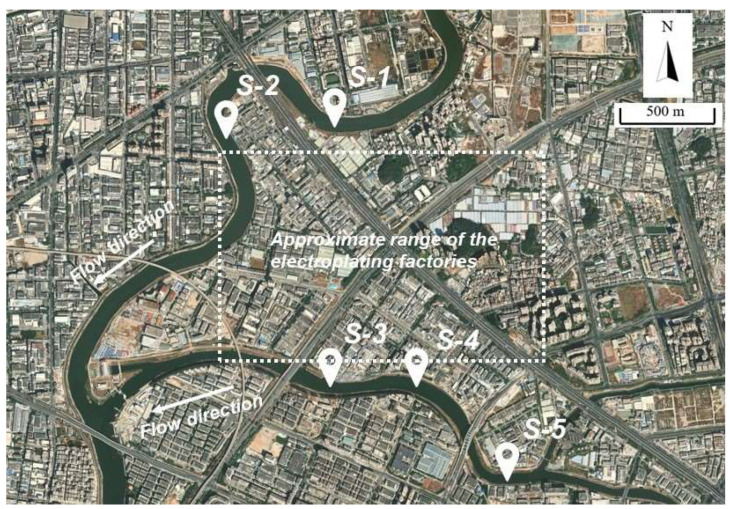
Sampling sites near the electroplating factories.

Water samples were collected from a river near electroplating factories in Shenzhen. S1 and S5 were located approximately 500 m upstream of the factories and S2, S3, and S4 were located downstream. The locations of the water sampling points around the plant are shown in Figure 1. Each water sample was collected in a polypropylene sample bottle and stored at 4 °C in a sampling box. Upon arrival at the laboratory, 500 mL of each water sample was filtered through a glass microfiber filter (GFF: diameter 150 mm; pore size 0.7 μm). The pH of the water was adjusted to 3.0 with a hydrochloric acid solution. The samples were then stored at 4 °C in the laboratory.

The water samples were extracted following a previously established method [29] with a few modifications. Briefly, Oasis WAX cartridges were preconditioned with 4 mL of 0.1% NH_4_OH/MeOH, 4.0 mL of methanol, and 4.0 mL of deionized water. Each filtrate was passed through a preconditioned cartridge at a flow rate of 5–10 mL min^−1^. The cartridge was then washed with 4 mL of deionized water and 25 mM of an acetic acid–ammonium acetate buffer solution (pH = 4). The WAX cartridges were placed in a centrifuge tube and centrifuged at 3000 rpm for 2 min to remove the excess water. An elution was carried out with 4 mL of methanol and 4 mL of 0.1% NH_4_OH/MeOH. The eluent was evaporated to dryness under a gentle N_2_ stream in a water bath at 40 °C, redissolved in 1.0 mL of methanol, transferred to a liquid chromatography (LC) vial, and evaporated to dryness under a gentle N_2_ stream. Each sample was reconstituted with methanol (0.5 mL) and spiked with an internal standard (0.5 ng).

Ultra-high-performance liquid chromatography combined with Q-Exactive Orbitrap Tandem Mass Spectrometry (UPLC-Q-Exactive MS) was applied for the non-target screening of the PFASs and 19 certified standards (Table S1) were applied for the further quantification of the PFASs. An RRHD Extend-C_18_ column (2.1 mm × 50 mm, 1.8 μm, Agilent) was used for separation. Mobile phase A consisted of 2 mM ammonium acetate/water, and mobile phase B consisted of methanol. The elution gradient was set as follows: 5–35% B for 1 min; 35–55% B for 7 min; 55–95% B for 17 min and maintained at 18 min; then back to the initial conditions (95% A) for 18.1 min and maintained at 20 min. The flow rate was set at 0.25 mL/min and the column oven was maintained at 35 °C. A 5 μL aliquot was injected into the LC-Q-Exactive MS system. The mass spectrometer was operated in the negative electrospray ionization in full scan mode (m/z 100–1000) (Table S1). The chromatograms are shown in Figure S1. The exact mass of the PFASs was applied to the screening and quantification of the PFASs.

All target analytes were quantified using an internal standard calibration curve (r > 0.99). The method reproducibility was evaluated based on the relative standard deviation (RSD) of the recovery of the spiked replicates. The limits of detection (LOD) were estimated based on signal-to-noise ratios of 3:1. The mean procedural recovery of the PFASs ranged from 81 to 122% and the LODs of the PFASs were 1–70 ng/L (Table S1). One procedural blank and one procedural recovery sample were also analyzed for each batch of samples to check for laboratory contamination and accuracy.

### 2.3. Ecological Risk Characterization

RQ methods were used in this study to roughly characterize the estimated ecological risks posed by the PFASs (as shown in Equation (1)). The ecological risks could be divided into four grades: high risk (RQ ≥ 1); medium risk (1 > RQ ≥ 0.1); low risk (0.1 > RQ ≥ 0.01); and no risk (RQ < 0.01) [30].


(1) $$\mathrm{RQ}=\frac{\mathrm{MEC}}{\mathrm{PNEC}}.$$

The PNEC values were extrapolated by SSDs. The log normal parametric fitting method was used for the construction of the SSD curves. The cumulative distribution function (CDF) is shown in Equation (2). The threshold concentration for protecting 95% of the species (i.e., the hazardous concentration for 5% of species, HC_5_) was obtained from the constructed SSD curve. The PNEC values were obtained using Equation (3). The model construction and related statistical calculations were completed using R (https://r-project.org (accessed on 2 July 2021)) and related packages such as “ssdtools” (https://bcgov.github.io/ssdtools/ (accessed on 2 July 2021)). The goodness-of-fit test for the normal distribution of the toxicity data was conducted using the Anderson–Darling test, Kolmogorov–Smirnov test, or Cramér–von Mises test.

(2) $$\mathrm{CDF}=(x,\mu ,\sigma )\frac{1}{2}+\frac{1}{2}\mathrm{erf}[\frac{\ln\;x-\mu }{\sqrt{2}\sigma }].$$

(3) $$\mathrm{PNEC}=\frac{{\mathrm{HC}}_{5}}{\mathrm{AF}}$$

where AF is the assessment factor, which was set to 5 in this study [6].

## 3. Results and Discussion

### 3.1. Predicted Toxicity Data by QSAR–ICE Models

The acute toxicity of PFASs to *Pseudokirchneriella subcapitata*, *Chlorella vulgaris*, *Daphnia magna*, and *Danio rerio* were collected (Table S2). There were 14 EC_50_ values (from 2.1 to 1130 mg/L) for *Pseudokirchneriella subcapitata*, 10 EC_50_ values (from 3.9 to 4030 mg/L) for *Chlorella vulgaris*, 10 LC_50_ values (from 0.06 mg/L to 2.58 × 10^5^ mg/L) for *Daphnia magna*, and 12 LC_50_ values (from 8.4 to 1500 mg/L) for *Danio rerio*. Based on the collected data, the calculated molecular descriptors (Supplementary Excel file), and a stepwise multiple linear regression, QSAR models for the four species were constructed (Equations (4)–(7) in Table 2). The four QSAR models were validated using the conventional correlation coefficient (r^2^) and the leave-one-out cross-validation correlation coefficient (q^2^) (Table 2). Generally, QSAR models with r^2^ > 0.6 and q^2^ > 0.5 can be regarded as having a relatively good predictive ability [31]. In this study, although the QSAR models showed only passable fitting degrees (R^2^) due to the relatively small datasets (*n*), the r^2^ and q^2^ values were >0.6 and >0.5, respectively, indicating that the established QSAR models had a good prediction ability and statistical significance (*p* < 0.05).

The molecular descriptors of the established models practicably explained the mechanism of acute toxicity (MOA). There was a positive correlation between the log EC_50_ and the total energy (TE), which is a molecular descriptor related to the molecular energies and stabilities of PFASs. These include molecular internal energy, translational kinetic energy, the energy of electrons in a molecule, the vibration energy between atoms in a molecule, and the energy of a molecule rotating around the center of a mass. A higher TE value indicates that the molecule is not easily polarized or absorbed by cells, thus resulting in a lower toxicity [32]. There was a positive correlation between the log EC_50_ and the lowest unoccupied molecule orbital energy (ELUMO). As the electronegativity of the F atom is the strongest, the PFASs reacted with the action site of the target organism as the electron acceptor. According to the frontier orbital theory, the occurrence of the reaction is related to the difference between the highest occupied orbital energy (EHOMO) of the electron donor and the ELUMO of the electron acceptor; that is, EHOMO–ELUMO (also known as the energy band gap). The larger the band gap, the easier the reaction and the stronger the binding force between the electron donor and the electron acceptor. Hence, the larger the band gap, the more obvious the toxicity and the lower the log LC_50_ value [33]. There was a negative correlation between the log LC_50_ and the nuclear–nuclear repulsive energy (ECCR). The electron cloud of atoms in a molecule is deformed more easily with an increase in the ECCR value, which makes PFASs more likely to polarize and enter a cell [33]. The log EC_50_ was negatively correlated with the octanol–water partition coefficient (*K*_ow_), which is related to the lipophilicity of PFASs. With an increase in the *K*_ow_ value, PFASs accumulate more easily in an organism, thus corresponding with a higher toxicity. *K*_ow_ is a key physico-chemical parameter serving as a classic molecular descriptor in QSAR modeling [34]. In this study, all 4 QSAR models contained *K*_ow_ (or log *K*_ow_), indicating the universality of *K*_ow_ in predicting aquatic acute toxicity. Moreover, it has been shown that *K*_ow_ is also important in applying QSAR models to predict toxicity in rodents [35,36] and in vitro toxicity assays [37,38]. In the practice of chemical management, *K*_ow_ can be used to justify waiving ecotoxicity tests (if log *K*_ow_ < 3) to assess bioaccumulation (if log *K*_ow_ < 3, the chemical can be considered to be non-bioaccumulative) [34]. As a result, *K*_ow_-based QSAR modeling can be an effective tool for predicting the toxicity of different endpoints in screening levels.

The acute toxicity data of perfluorobutyric acid (PFBA), PFOA, perfluorobutanesulfonic acid (PFBS), perfluorohexanesulfonic acid (PFHxS), PFOS, and 6:2 chlorinated polyfluoroalkyl ether sulfonate (6:2 Cl-PFESA) for the four selected species were predicted using the four QSAR models, as shown in Table 3. The results predicted by the QSAR models showed that the toxicities of novel PFASs or substitutes such as 6:2 Cl-PFESA, PFBA, and PFBS were higher than those of PFOS and PFOA. The insertion of an oxygen atom into the 6:2 Cl-PFESA molecule could increase the activity of the molecule. The experimental results of other studies have also indicated that the presence of oxygen atoms could increase the toxicity of PFASs [39]. Due to their smaller molecular weight, short-chain PFAS substitutes (e.g., PFBA and PFBS) may be more easily polarized and absorbed by cells, thus increasing toxicity.

Based on the measured toxicity data collected from databases (Table S3) and the predicted data of the QSAR models, the 13, 34, 17, 13, 13, and 13 ICE models available for PFBA, PFOA, PFBS, PFHxS, PFOS, and 6:2 Cl-PFESA (Table S4) were selected for their toxicity extrapolation, respectively [28]. The acute toxicity data estimated by the QSAR–ICE models constructed for the above six substances are listed in Table S5.

### 3.2. Calculation and Comparison of the PNEC Values of SSDs Produced Using Predicted and Measured Data

**Figure 2:**
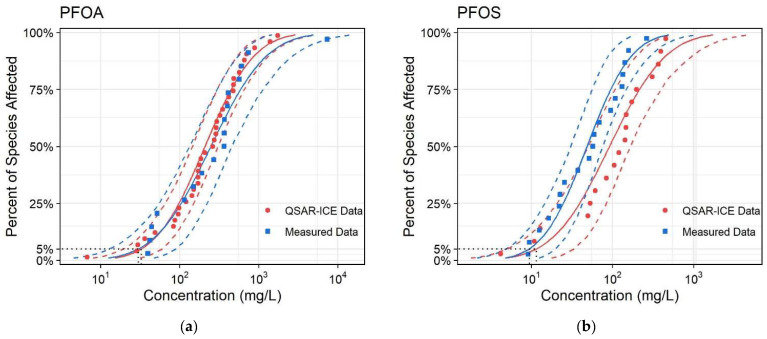

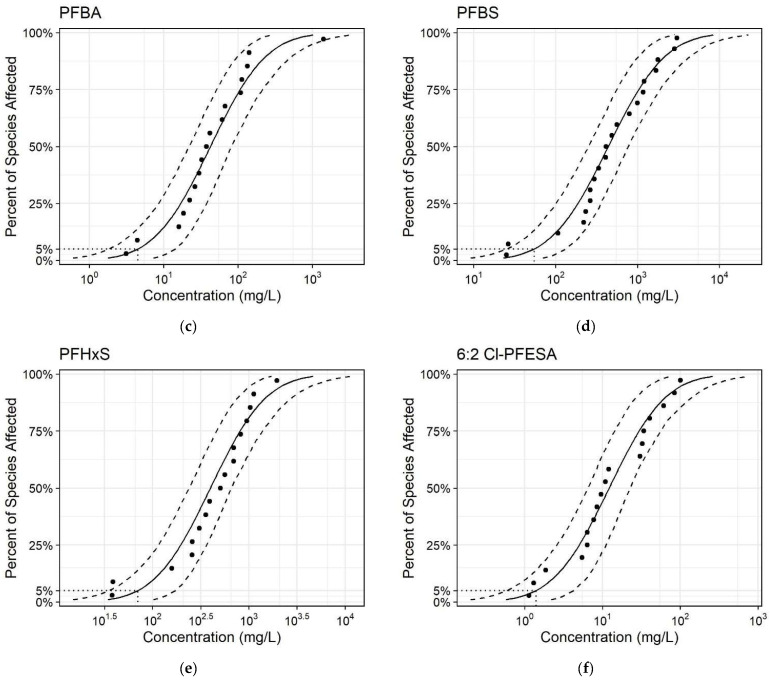
SSD curves based on the predicted data (six PFASs) and measured data (PFOA and PFOS). (**a**) PFOA (**b**) PFOS (**c**) PFBA (**d**) PFBS (**e**) PFHxS (**f**) 6:2 Cl-PFESA.

Figure 2 shows the SSD curves based on the predicted data by the QSAR–ICE models (the six PFASs) and measured data, respectively. The results of the goodness-of-fit tests for the acute toxicity data are shown in Table S6. The results of the goodness-of-fit tests for all six PFASs were less than the corresponding thresholds, indicating that the obtained acute toxicity data were consistent with the log normal distribution. The HC_5_ and PNEC values are presented in Table 4. The order of the HC_5_ values was ranked from low to high, which was 6:2 Cl-PFESA < PFBA < PFOS < PFOA < PFBS < PFHxS. The measured acute toxicity data used in the SSD curves of PFOA and PFOS are shown in Table S7. The HC_5_ values obtained by the two methods were compared in order to evaluate the accuracy of the QSAR–ICE–SSD models. As shown in Table 4, the HC_5_ values of the QSAR–ICE–SSD models were 1.16 times (PFOA) and 1.20 times (PFOS) higher than the calculated values based on the measured toxicity data. As a result, the QSAR–ICE–SSD models had a certain reliability for predicting PNEC values when limited data were available.

### 3.3. Concentrations of PFASs in the River near the Electroplating Factories

**Figure 3:**
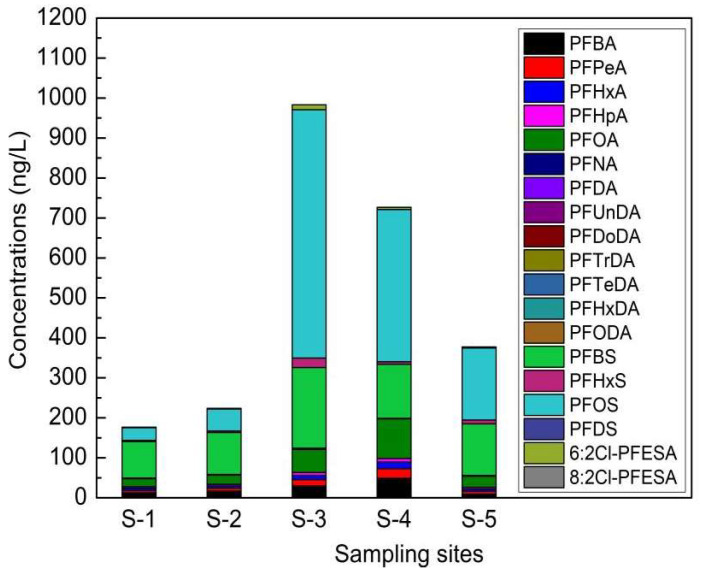
PFAS concentrations in a river near electroplating factories.

The Σ_19_PFAS concentrations ranged from 177 to 983 ng/L in the river water samples (Figure 3); the mean values of PFOS, PFBS, and PFHxS were 254, 132, and 9.18 ng/L, respectively. One study on PFASs in 28 rivers in eastern China showed that the PFAS concentration ranges were 39–212 ng/L and 0.68–146 ng/L in Shanghai and Zhejiang Province, respectively [40]. Another study of fluoropolymer facilities showed that PFAS concentrations ranged from 0.96 to 4534.41 ng/L in nearby rivers [41]. Industrial processes involving the use of PFASs are a conspicuous source of PFASs for the environment. Major downstream industrial users, such as electroplating facilities, have started to use alternatives [42].

### 3.4. Ecological Risks of PFASs

Based on the monitoring data of PFASs in this study, PFBA, PFOA, PFBS, PFHxS, PFOS, and 6:2 Cl-PFESA were the mainly detected PFASs in the river near the electroplating facilities. The PNEC values of the six typical PFASs were calculated using the QSAR–ICE–SSD models. The RQ values of the PFASs in this study and in four other electroplating areas in Guangdong Province in China [43] were then calculated, as listed in Table 5. The results showed that the six PFASs posed no ecological risks to the river although, compared with other electroplating areas, the RQ values of PFOA, PFBS, PFOS, and 6:2 Cl-PFESA were higher in this study. Only ecological risks based on acute PNEC values were calculated due to limited data. However, it has been suggested that PFASs may have reproductive and growth adverse effects on aquatic organisms [12]. PFASs are persistent, bioaccumulative, and can be transported long distances, thus causing lasting damage to the aquatic organism [12]. The ecological risks of PFASs in this study may have been underestimated.

### 3.5. Implications and Limitations

It has been shown that there are around 4700 PFASs on the global market [26]. The development of rapid in silico methods avoiding time-consuming and laborious animal experiments is necessary. QSAR–ICE–SSD models can be used to derive screening-level PNEC values in both prospective and retrospective assessments for novel PFASs where ecotoxicity data are lacking. The acute toxicity data of at least 15 species covering three trophic levels of an ecosystem can be derived [26]. These data can meet the requirements of the minimum datasets for the construction of SSD models, improving ecological relevance and reducing the uncertainty caused by the limited data quantity [3,6,44]. We selected as much as possible of the acute toxicity data of four model species from a wide range of PFAS groups (e.g., PFCAs, PFSAs, PFESAs) selected for QSAR modeling. The selected ICE models were also developed from data containing PFOS. This improved the adaptability and reliability of the model, reducing the uncertainty caused by the construction of the models [45]. As mentioned in Section 3.1, *K*_ow_ was found to be a key molecular descriptor in predicting aquatic acute toxicity in QSAR modeling. A possible future research direction could be to identify the role of *K*_ow_ in QSAR modeling to predict other endpoints (e.g., no observed adverse effect level (NOAEL), benchmark dose (BMD) of acute toxicity in rodents, or in vitro toxicity assays). This can help us understand the MOA of PFASs and integrate the data between an ERA and a human health risk assessment [46].

A major limitation of this study was that the acute toxicity data used to develop the QSAR model for each species was quite limited. Correspondingly, the small data sizes led to a just passable fitting effect of the QSAR models and limited the use of machine learning algorithms such as random forest [47,48]. One possible improvement of this issue is the selection of more acute toxicity data in QSAR modeling from not only PFASs but also other organic chemicals based on the same MOA [49]. However, this method is based on a sufficient understanding of the MOA of PFASs, in which further study is needed [26]. Another limitation of the QSAR–ICE–SSD approach was that only acute toxicity data were used and only acute PNEC values could be derived. Given the current limited availability of chronic (e.g., growth and reproductive effect) no observed effect concentration (NOEC), lowest observed effect concentration (LOEC), and 10% effect concentration (EC_10_) data, it was not possible to follow our approach to derive chronic PNEC values. Using an acute-to-chronic ratio to extrapolate the chronic toxicity data for each species is a possible way; however, it can increase the uncertainty of the data quality [50].

## 4. Conclusions

In summary, the QSAR–ICE–SSD models predicted the following HC_5_ values for six PFASs: 0.804 mg/L (PFBA), 6.27 mg/L (PFOA), 10.1 mg/L (PFBS), 12.9 mg/L (PFHxS), 2.09 mg/L (PFOS), and 0.254 mg/L (6:2 Cl-PFESA). The Σ_19_PFAS concentrations were 177–983 ng/L in the nearby river of electroplating factories in Shenzhen. The results indicated that these electroplating factories may not be the source of the PFASs in the local aquatic environment. The RQ values of the six PFASs ranged from 2.29 × 10^−7^ to 2.97 × 10^−4^ in the nearby river.

## Figures and Tables

**Table 1:** Molecular descriptors used in this study.

No.	Molecular Descriptors	Abbreviations	Units
1	Heat of formation	HOF	kcal/mol
2	Total energy	TE	EV
3	Electronic energy	EE	EV
4	Core–core repulsion energy	ECCR	EV
5	COSMO area	CA	Å2
6	COSMO volume	CV	Å3
7	Gradient norm	GN	-
8	Gradient norm per atom	GN *p* A	-
9	Ionization potential	IP	EV
10	Lowest unoccupied molecule orbital energy	ELUMO	EV
11	Highest occupied molecular orbital energy	EHOMO	EV
12	Molecular weight	MW	-
13	Octanol–water partition coefficient	*K*_ow_	-

**Table 2:** QSAR models and their validation parameters.

Species	Models	Equations	*n* ^a^	R^2^ ^b^	r^2 c^	q^2 d^	*p* ^e^
*Pseudokirchneriella subcapitata*	log EC_50_ = −log *K*_ow_ × 8.82 + TE × 47.8 + log E_LUMO_ × 1.47 − E_CCR_ × 39.7 + 50.3	(4)	14	0.770	0.742	0.701	0.006
*Chlorella vulgaris*	log EC_50_ = −4.18 × *K*_ow_ − 0.332 × E_CCR_ − 4.29	(5)	10	0.592	0.751	0.673	0.043
*Daphnia magna*	log LC_50_ = −*K*_ow_ × 4.09 + log TE × 9.75 − E_CCR_ × 7.03 + log E_LUMO_ × 1.63 + 1.95	(6)	10	0.370	0.605	0.580	0.045
*Danio rerio*	log LC_50_ = −*K*_ow_ × 1.03 − E_CCR_ × 1.04 + E_LUMO_ × 0.318 + 2.94	(7)	12	0.558	0.722	0.630	0.046

^a^ *n*: number of toxicity data. ^b^ R^2^: coefficient of determination of the multiple regression. ^c^ r^2^: conventional correlation coefficient or non-validation correlation coefficient. ^d^ q^2^: leave-one-out cross-validation correlation coefficient. ^e^ *p*: statistical significance.

**Table 3:** Predicted acute toxicity data (mg/L) of six PFASs by QSAR models.

PFASs	CAS No.	*Pseudokirchneriella* *subcapitata*	*Chlorella vulgaris*	*Daphnia magna*	*Danio rerio*
PFBA	375-22-4	67.1	112	37.4	1410
PFOA	335-67-1	478	150	570	98.5
PFBS	375-73-5	2840	222	487	1000
PFHxS	355-46-4	1030	258	821	256
PFOS	1763-23-1	53	309	173	61.3
6:2 Cl-PFESA	73606-19-6	1.3	84.9	10.9	32.7

**Table 4:** The HC_5_ and PNEC values based on the predicted data (six PFASs) and measured data (PFOA and PFOS).

	PFBA	PFOA	PFOA (Measured)	PFBS	PFHxS	PFOS	PFOS (Measured)	6:2 Cl-PFESA
HC_5_ (mg/L)	4.02	31.4	27	50.5	64.5	10.5	8.72	1.27
PNEC (mg/L)	0.804	6.27	-	10.1	12.9	2.09	-	0.254

**Table 5:** RQ values (×10^−6^) of six PFASs in this study and in the literature.

Sample Sites	RQ Values	PFBA	PFOA	PFBS	PFHxS	PFOS	6:2 Cl-PFESA
This Study	Range	11.5–60.9	3.25–15.8	9–20	0.23–1.83	15.3–297	5.1–49.8
	Mean	29.1	7.26	13.1	0.71	121	19.1
Gaoping [43]	Range	2.44–24.1	0.18–2.81	0–0.6	0.04–0.48	0–7.66	0–1.38
	Mean	11.1	0.57	0.21	0.23	1.07	0.12
Humen [43]	Range	2.44–42.3	0.53–3.24	0.13–1.24	0–27,000	0–4.41	0–3.82
	Mean	24.7	1.42	0.64	9290	1.29	0.39
Boluo [43]	Range	16.2–54.3	0.064–2.99	0–14.9	0–1.87	0–15.6	0–1.26
	Mean	24.3	0.76	4.17	0.31	2.03	0.16
Shatian [43]	Range	22–105	0.99–6.98	0.23–4.7	0–0.3	0–6.44	0
	Mean	38.9	3.4	1.7	0.2	1.57	0

## Data Availability

Not applicable.

## Supplementary Materials

The following are available online: Supplementary Excel file; Table S1: PFAS properties and m/z values for quantification; Table S2: The collected acute toxicity of PFASs to four species for the QSAR models; Table S3: The collected acute toxicity of PFASs for the ICE models; Table S4: The ICE models used in this study; Table S5: The predicted acute toxicity of PFASs by QSAR–ICE models; Table S6: The results of the goodness-of-fit tests of the SSD models; Table S7: The measured acute toxicity of PFOA and PFOS; Figure S1: Liquid chromatography coupled to hybrid quadrupole-Orbitrap mass spectrometer LC-MS (Q-Exactive) (Thermo fisher scientific, USA) chromatograms for (A) standard PFASs and their (B) internal standard.
