# Supplementary material for: A QSAR–ICE–SSD Model Prediction of the PNECs for Per- and Polyfluoroalkyl Substances and Their Ecological Risks in an Area of Electroplating Factories
Source: Molecules. 2021 Oct 30;26(21):6574. doi: 10.3390/molecules26216574 (PMC8587016; doi:10.3390/molecules26216574)
Supplement: Supplementary file 1 [file molecules-26-06574-s001.zip › molecules-1434437-supplementary.pdf]

molecules

Support Information

A QSAR–ICE–SSD Model Prediction of the PNECs for Per- and Polyfluoroalkyl Substances and Their Ecological Risks in an Area of Electroplating Factories

Jiawei Zhang [1,2], Mengtao Zhang [1], Huanyu Tao [1,2], Guanjing Qi [1], Wei Guo [1,3], Hui Ge [1,*] and Jianghong Shi [1,*]

[1] State Environmental Protection Key Laboratory of Integrated Surface Water-Groundwater Pollution Control, School of Environmental Science and Engineering, Southern University of Science and Technology, Shenzhen 518055, China; 11750007@mail.sustech.edu.cn (J.Z.); 11652003@mail.sustech.edu.cn (M.Z.); 11850006@mail.sustech.edu.cn (H.T.); 11849066@mail.sustech.edu.cn (G.Q.); guow@sustech.edu.cn (W.G.)
[2] Environmental Engineering Research Centre, Department of Civil Engineering, The University of Hong Kong, Hong Kong, China
[3] Key Laboratory of Beijing for Water Quality Science and Water Environment Recovery Engineering, Beijing University of Technology, Beijing 100124, China
* Correspondence: geh@sustech.edu.cn (H.G.); shijh@sustech.edu.cn (J.S.)

Table S1. PFASs properties and m/z values for quantification.

Target coumpounds	Molecular formula	CAS number	m/z	Retention time (min)	LOD	Recoveries
PFBA	$C_4HF_7O_2$	375-22-4	212.9792	2.9	3.0	109%
PFPeA	$C_5HF_9O_2$	2706-90-3	262.976	4.9	3.0	106
PFHxA	$C_6HF_{11}O_2$	307-24-4	312.97281	7.5	25.0	104%
PFHpA	$C_7HF_{13}O_2$	375-85-9	362.96962	9.7	5.0	108%
PFOA	$C_8HF_{15}O_2$	335-67-1	412.96643	11.4	5.0	108%
PFNA	$C_9HF_{17}O_2$	375-95-1	462.96323	12.6	10.0	100%
PFDA	$C_{10}HF_{19}O_2$	335-76-2	512.96004	13.6	10.0	99%
PFUnDA	$C_{11}HF_{21}O_2$	2058-94-8	562.95684	14.4	20.0	109%
PFDoDA	$C_{12}HF_{23}O_2$	307-55-1	612.95365	15.1	20.0	100%
PFTrDA	$C_{13}HF_{25}O_2$	72629-94-8	662.95046	15.7	25.0	81%
PFTeDA	$C_{14}HF_{27}O_2$	376-06-7	712.94726	16.2	20.0	104%
PFHxDA	$C_{16}HF_{31}O_2$	67905-19-5	812.94088	17	40.0	122%
PFODA	$C_{18}HF_{33}O_2$	16517-11-6	912.93449	17.7	70.0	120%
PFBS	$C_4F_9O_3SNa$	375-73-5	298.94299	5.6	4.0	98%
PFHxS	$C_6F_{13}SO_3Na$	355-46-4	398.9366	10	4.0	102%
PFOS	$C_8F_{17}SO_3Na$	1763-23-1	498.93022	12.6	1.0	100%
PFDS	$C_{10}F_{21}SO_3Na$	335-77-3	598.92383	14.3	10.0	100%
6:2 Cl-PFESA	$C_8F_{16}ClSO_4K$	73606-19-6	530.8945	13	8.0	84%
8:2 Cl-PFESA	$C_{10}F_{20}ClSO_4K$	83329-89-9	630.8881	14.5	11.0	85%
$[^{13}C_2]$-PFHxA	$[^{13}C_2]$-PFHxA	/	314.9795	7.61		
$[^{13}C_2]$-PFOA	$[^{13}C_4]$-PFOA	/	416.9798	11.52		
$[^{13}C_5]$-PFNA	$[^{13}C_5]$-PFNA	/	467.98	12.7		
$[^{13}C_2]$-PFDA	$[^{13}C_2]$-PFDA	/	514.9667	13.69		
$[^{13}C_2]$-PFUnDA	$[^{13}C_2]$-PFUnDA	/	564.9635	14.51		
$[^{13}C_2]$-PFDoDA	$[^{13}C_2]$-PFDoDA	/	614.9603	15.18		
$[^{18}O_2]$-PFHxS	$[^{13}O_2]$-PFHxS	/	402.945	10.15		
$[^{13}C_4]$-PFOS	$[^{13}C_4]$-PFOS	/	502.9436	12.74		

Table S2. The collected acute toxicity of PFASs to four species for the QSAR models.

Species	No.	CAS No.	PFASs	EC_{50}/LC_{50} (mg/L)	References
Pseudo-kirchneri-ella subcap-itata	1	335-67-1	Perfluorooctanoic acid	246	[1-3]
	2	2923-18-4	Sodium trifluoroacetate	13.7	[4]
	3	914637-49-3	2H,2H,3H,3H-Perfluorooctanoic acid	22.5	[5]
	4	2706-90-3	Perfluoropentanoic acid	90.0	[5]
	5	375-22-4	Perfluorobutanoic acid	262	[1]
	6	375-95-1	Perfluorononanoic acid	482	[1]
	7	335-76-2	Perfluorodecanoic acid	10.6	[5]
	8	2795-39-3	Potassium perfluorooctanesulfonate	139	[6]
	9	27854-31-5	2-Perfluorooctyl ethanoic acid	5.00	[5]
	10	53826-12-3	2-Perfluorohexyl ethanoic acid	47.9	[5]
	11	812-70-4	3-Perfluoroheptylpropanoic acid	2.10	[5]
	12	70887-84-2	2H-Perfluoro-2-decenoic acid	7.50	[5]
	13	355-80-6	1H,1H,5H-Perfluoropentanol	1130	[1]
	14	70887-88-6	2H-Perfluoro-2-octenoic acid	28.5	[5]
Chlorella vulgaris	1	335-67-1	Perfluorooctanoic acid	977	[7]
	2	307-24-4	Perfluorohexanoic acid	4030	[7]
	3	375-95-1	Perfluorononanoic acid	497	[7]
	4	2795-39-3	Potassium perfluorooctanesulfonate	84.8	[8]
	5	375-85-9	Perfluoroheptanoic acid	1900	[7]
	6	27854-31-5	2-Perfluorooctyl ethanoic acid	5.90	[9]
	7	53826-12-3	2-Perfluorohexyl ethanoic acid	26.2	[9]
	8	70887-88-6	2H-Perfluoro-2-octenoic acid	31.8	[9]
	9	53826-13-4	2-Perfluorodecyl ethanoic acid	4.20	[9]
	10	70887-94-4	2H-Perfluoro-2-dodecenoic acid	3.90	[9]
Daphnia magna	1	335-67-1	Perfluorooctanoic acid	202	[10]
	2	335-24-0	Potassium perfluoro-p-ethylcyclohexanesulfonate	187	[11]
	3	375-95-1	Perfluorononanoic acid	80.9	[12]
	4	2795-39-3	Potassium perfluorooctanesulfonate	78.1	[10]
	5	45298-90-6	Perfluorooctanesulfonate	49.3	[12]
	6	335-76-2	Perfluorodecanoic acid	2.58×10^{5}	[13]
	7	27854-31-5	2-Perfluorooctyl ethanoic acid	3.52	[14]
	8	70887-84-2	2H-Perfluoro-2-decenoic acid	8.45	[14]

	9	53826-13-4	2-Perfluorodecyl ethanoic acid	0.06	[14]
	10	70887-94-4	2H-Perfluoro-2-dodecenoic acid	0.35	[14,15]
Danio rerio	1	307-24-4	Perfluorohexanoic acid	91.1	[16]
	2	335-67-1	Perfluorooctanoic acid	210	[17-21]
	3	375-95-1	Perfluorononanoic acid	128	[17-19]
	4	335-76-2	Perfluorodecanoic acid	8.40	[21]
	5	375-73-5	Perfluorobutanesulfonic acid	1500	[21]
	6	355-46-4	Perfluorohexanesulfonic acid	136	[16]
	7	1763-23-1	Perfluorooctanesulfonic acid	64.2	[18,22-24]
	8	2795-39-3	Potassium perfluorooctanesulfonate	21.8	[25-28]
	9	30381-98-7	Ammonium bis(N-ethyl-2-perfluorooctylsulfonaminoethyl)phosphate	211	[18]
	10	73606-19-6	Potassium 9-chlorohexadecafluoro-3-oxanonane-1-sulfonate	13.8	[29]
	11	647-42-7	6:2 Fluorotelomer alcohol	302	[16]
	12	34455-29-3	6:2 Fluorotelomer sulfonamide betaine	57.4	[29]

Table S3. The collected acute toxicity of PFASs for the ICE models.

PFASs	No.	Species	LC_{50}/EC_{50} (mg/L)	References
	1	*Carassius auratus*	607	[10]
PFOA	2	*Pseudorasbora parva*	365	[10]
	3	*Limnodrilus hoffmeisteri*	568	[10]
PFOS	1	*Myriophyllum spicatum*	12.5	[30]
PFBS	1	*Lepomis macrochirus*	6450	[31]

Table S4. The ICE models used in this study [32].

**No.**	**Surrogate species**	**Predicted species**	**ICE models**	**Application**
1	*Chlorella vulgaris*	*Scenedesmus obliquus*	y=1.00x+0.0644	Six PFASs
2	*Daphnia magna*	*Ceriodaphnia dubia*	y=1.02x-0.186	Six PFASs
3	*Daphnia magna*	*Chironomus plumosus*	y=0.743x+0.808	Six PFASs
4	*Daphnia magna*	*Daphnia pulex*	y=0.994x-0.124	Six PFASs
5	*Daphnia magna*	*Eriocheir sinensis*	y=0.676x+2.06	Six PFASs
6	*Daphnia magna*	*Gammarus lacustris*	y=0.697x+0.458	Six PFASs
7	*Daphnia magna*	*Gammarus pulex*	y=1.01x-0.278	Six PFASs
8	*Daphnia magna*	*Lymnaea stagnalis*	y=1.01x-0.0066	Six PFASs
9	*Daphnia magna*	*Macrobrachium nipponense*	y=0.816x-0.24	Six PFASs
10	*Daphnia magna*	*Macrobrachium rosenbergii*	y=0.990x-0.261	Six PFASs
11	*Daphnia magna*	*Paratanytarsus dissimilis*	y=0.572x+2.17	Six PFASs
12	*Daphnia magna*	*Paratanytarsus parthenogeneticus*	y=0.938x+0.743	Six PFASs
13	*Daphnia magna*	*Physa gyrina*	y=0.991x-0.021	Six PFASs
14	*Carassius auratus*	*Ctenopharyngodon idellus*	y=0.798x+0.705	PFOA
15	*Carassius auratus*	*Euphlyctis hexadactylus*	y=1.15x-0.775	PFOA
16	*Carassius auratus*	*Gobiocypris rarus*	y=0.893x+0.293	PFOA
17	*Carassius auratus*	*Hypophthalmichthys molitrix*	y=0.977x-0.026	PFOA
18	*Carassius auratus*	*Ictalurus punctatus*	y=0.907x+0.268	PFOA
19	*Carassius auratus*	*Lepomis cyanellus*	y=0.850x+0.0071	PFOA
21	*Carassius auratus*	*Lepomis macrochirus*	y=0.957x-0.267	PFOA
22	*Carassius auratus*	*Micropterus salmoides*	y=0.869x-0.465	PFOA
23	*Carassius auratus*	*Misgurnus anguillicaudatus*	y=0.950x+1.41	PFOA
24	*Carassius auratus*	*Monopterus albus*	y=0.792x+0.84	PFOA
25	*Carassius auratus*	*Oncorhynchus mykiss*	y=1.01x-0.594	PFOA
26	*Carassius auratus*	*Pelteobagrus fulvidraco*	y=0.439x+1.29	PFOA
27	*Carassius auratus*	*Poecilia reticulata*	y=0.735x+0.372	PFOA
28	*Carassius auratus*	*Pseudorasbora parva*	y=1.03x-0.0976	PFOA
29	*Carassius auratus*	*Rana limnocharis*	y=0.581x+1.33	PFOA
30	*Pseudorasbora parva*	*Carassius auratus*	y=0.784x+0.797	PFOA

31	*Pseudorasbora parva*	*Cyprinus carpio*	y=0.981x-0.517	PFOA
32	*Limnodrilus hoff-meisteri*	*Branchiura sowerbyi*	y=0.841x+0.393	PFOA
33	*Limnodrilus hoff-meisteri*	*Lumbriculus variegatus*	y=0.980x-0.404	PFOA
34	*Limnodrilus hoff-meisteri*	*Tubifex tubifex*	y=1.05x-0.256	PFOA
35	*Lepomis macro-chirus*	*Carassius auratus*	y=0.752x+1.13	PFBS
36	*Lepomis macro-chirus*	*Ictalurus punctatus*	y=0.788x+0.862	PFBS
37	*Lepomis macro-chirus*	*Oncorhynchus mykiss*	y=0.943x+0.0589	PFBS
38	*Lepomis macro-chirus*	*Poecilia reticulata*	y=0.748x+0.976	PFBS
39	*Myriophyllum spi-catum*	*Ceratophyllum demersum*	y=0.941x-0.235	PFOS

Table S5. The predicted acute toxicity of PFASs by QSAR-ICE models.

PFASs	No.	Species	LC_{50}/EC_{50} (mg/L)
PFBA	1	*Ceriodaphnia dubia*	29.84
	2	*Chironomus plumosus*	15.96
	3	*Daphnia pulex*	26.24
	4	*Eriocheir sinensis*	140.31
	5	*Gammarus lacustris*	4.41
	6	*Gammarus pulex*	22.24
	7	*Lymnaea stagnalis*	107.97
	8	*Macrobrachium nipponense*	41.81
	9	*Macrobrachium rosenbergii*	3.11
	10	*Paratanytarsus dissimilis*	18.42
	11	*Paratanytarsus parthenogeneticus*	61.18
	12	*Physa gyrina*	32.46
	13	*Scenedesmus obliquus*	133.66
PFOA	1	*Branchiura sowerbyi*	170.89
	2	*Carassius auratus*	143.01
	3	*Ceriodaphnia dubia*	479.64
	4	*Chironomus plumosus*	120.64
	5	*Ctenopharyngodon idellus*	207.75
	6	*Cyprinus carpio*	87.00
	7	*Daphnia pulex*	393.01
	8	*Eriocheir sinensis*	884.26
	9	*Euphlyctis hexadactylus*	702.60
	10	*Gammarus lacustris*	29.47

	11	*Gammarus pulex*	349.76
	12	*Gobiocypris rarus*	287.02
	13	*Hypophthalmichthys molitrix*	420.63
	14	*Ictalurus punctatus*	324.76
	15	*Lepomis cyanellus*	83.67
	16	*Lepomis macrochirus*	186.19
	17	*Lumbriculus variegatus*	170.93
	18	*Lymnaea stagnalis*	658.72
	19	*Macrobrachium nipponense*	28.79
	20	*Macrobrachium rosenbergii*	273.04
	21	*Micropterus salmoides*	36.11
	22	*Misgurnus anguillicaudatus*	294.39
	23	*Monopterus albus*	262.41
	24	*Oncorhynchus mykiss*	172.89
	25	*Paratanytarsus dissimilis*	290.37
	26	*Paratanytarsus parthenogeneticus*	1391.20
	27	*Pelteobagrus fulvidraco*	6.74
	28	*Physa gyrina*	483.05
	29	*Poecilia reticulata*	95.70
	30	*Pseudorasbora parva*	1748.47
	31	*Rana limnocharis*	48.71
	32	*Scenedesmus obliquus*	178.68
	33	*Selenastrum bibraianum*	477.50
	34	*Tubifex tubifex*	593.94
	1	*Carassius auratus*	1776.50
	2	*Ceriodaphnia dubia*	408.24
	3	*Chironomus plumosus*	107.28
	4	*Daphnia pulex*	335.87
	5	*Eriocheir sinensis*	794.64
PFBS	6	*Gammarus lacustris*	26.40
	7	*Gammarus pulex*	298.07
	8	*Ictalurus punctatus*	1686.46
	9	*Lymnaea stagnalis*	561.30
	10	*Macrobrachium nipponense*	25.31
	11	*Macrobrachium rosenbergii*	233.49

	12	*Oncorhynchus mykiss*	3018.29
	13	*Paratanytarsus dissimilis*	265.28
	14	*Paratanytarsus parthenogeneticus*	1199.37
	15	*Physa gyrina*	412.97
	16	*Poecilia reticulata*	1172.93
	17	*Scenedesmus obliquus*	264.06
PFHxS	1	*Ceriodaphnia dubia*	694.99
	2	*Chironomus plumosus*	158.06
	3	*Daphnia pulex*	564.14
	4	*Eriocheir sinensis*	1130.70
	5	*Gammarus lacustris*	37.98
	6	*Gammarus pulex*	505.32
	7	*Lymnaea stagnalis*	951.93
	8	*Macrobrachium nipponense*	38.75
	9	*Macrobrachium rosenbergii*	391.40
	10	*Paratanytarsus dissimilis*	357.50
	11	*Paratanytarsus parthenogeneticus*	1957.19
	12	*Physa gyrina*	692.78
	13	*Scenedesmus obliquus*	307.10
PFOS	1	*Ceratophyllum demersum*	4.17
	2	*Ceriodaphnia dubia*	142.28
	3	*Chironomus plumosus*	49.78
	4	*Daphnia pulex*	120.22
	5	*Eriocheir sinensis*	395.10
	6	*Gammarus lacustris*	12.84
	7	*Gammarus pulex*	104.74
	8	*Lymnaea stagnalis*	197.10
	9	*Macrobrachium nipponense*	10.88
	10	*Macrobrachium rosenbergii*	83.90
	11	*Paratanytarsus dissimilis*	146.89
	12	*Paratanytarsus parthenogeneticus*	454.56
	13	*Physa gyrina*	148.19
	14	*Scenedesmus obliquus*	367.93
6:2 Cl-PFESA	1	*Ceriodaphnia dubia*	8.52
	2	*Chironomus plumosus*	6.40

3	*Daphnia pulex*	7.73
4	*Eriocheir sinensis*	61.12
5	*Gammarus lacustris*	1.87
6	*Gammarus pulex*	6.41
7	*Lymnaea stagnalis*	12.04
8	*Macrobrachium nipponense*	1.14
9	*Macrobrachium rosenbergii*	5.45
10	*Paratanytarsus dissimilis*	30.29
11	*Paratanytarsus parthenogeneticus*	34.06
12	*Physa gyrina*	9.59
13	*Scenedesmus obliquus*	100.82

Table S6. The results of the goodness-of-fit tests of SSD models.

SSD models	A-D Test	K-S Test	C-M Test
Threshold value (α=0.01)	1.04	1.04	0.179
PFBA	0.399	0.599	0.048
PFOA (QSAR-ICE data)	0.442	0.652	0.067
PFOA (Measured data)	0.599	0.630	0.078
PFBS	0.443	0.739	0.050
PFHxS	0.634	0.715	0.079
PFOS (QSAR-ICE data)	0.674	0.673	0.102
PFOS (Measured data)	0.349	0.513	0.048
6:2 Cl-PFESA	0.355	0.611	0.050

Table S7. The measured acute toxicity of PFOA and PFOS.

**PFASs**	**No.**	**Species**	**LC_{50}/EC_{50} (μg/L)**	**References**
	1	*Anabaena sp.*	3.95×10^4	[33,34]
	2	*Scenedesmus acutus*	4.40×10^4	[35]
PFOA	3	*Chlamydomonas reinhardtii*	5.19×10^4	[35]
	4	*Chlorella pyrenoidosa*	1.91×10^5	[3]
	5	*Scenedesmus quadricauda*	2.70×10^5	[10]

	6	*Skeletonema marinoi*	3.69×10^5	[7]
	7	*Brachionus calyciflorus*	1.50×10^5	[36]
	8	*Bufo gargarizans*	1.15×10^5	[10]
	9	*Carassius auratus*	6.07×10^5	[10]
	10	*Pimephales promelas*	4.13×10^5	[20]
	11	*Pseudorasbora parva*	3.65×10^5	[10]
	12	*Chironomus plumosus*	4.02×10^5	[10]
	13	*Daphnia magna*	7.37×10^6	[10,13]
	14	*Macrobrachium nipponense*	3.67×10^5	[10]
	15	*Cipangopaludina cathayensis*	7.40×10^5	[10]
	16	*Dugesia japonica*	4.21×10^4	[37]
	17	*Limnodrilus hoffmeisteri*	5.68×10^5	[10]
	1	*Isochrysis galbana*	3.75×10^4	[38]
	2	*Anabaena sp.*	1.63×10^4	[34]
	3	*Dugesia japonica*	2.56×10^4	[39]
	4	*Ligumia recta*	1.42×10^5	[40]
	5	*Lampsilis siliquoidea*	1.58×10^5	[40]
	6	*Pelophylax nigromaculatus*	9.50×10^4	[41]
	7	*Xenopus laevis*	5.15×10^4	[42]
	8	*Perna viridis*	6.83×10^4	[43]
	9	*Pagrus major*	2.26×10^4	[44]
PFOS	10	*Navicula pelliculosa*	2.63×10^5	[30]
	11	*Lemna gibba*	5.91×10^4	[30]
	12	*Daphnia pulicaria*	1.34×10^5	[30]
	13	*Anabaena flos-aquae*	1.31×10^5	[30]
	14	*Lemna gibba*	1.08×10^5	[30]
	15	*Myriophyllum spicatum*	1.25×10^4	[30]
	16	*Unio complanatus*	5.70×10^4	[30]
	17	*Artemia salina*	9.40×10^3	[30]
	18	*Rainbow trout*	2.20×10^4	[30]
	19	*Pimephales promelas*	9.10×10^3	[30]

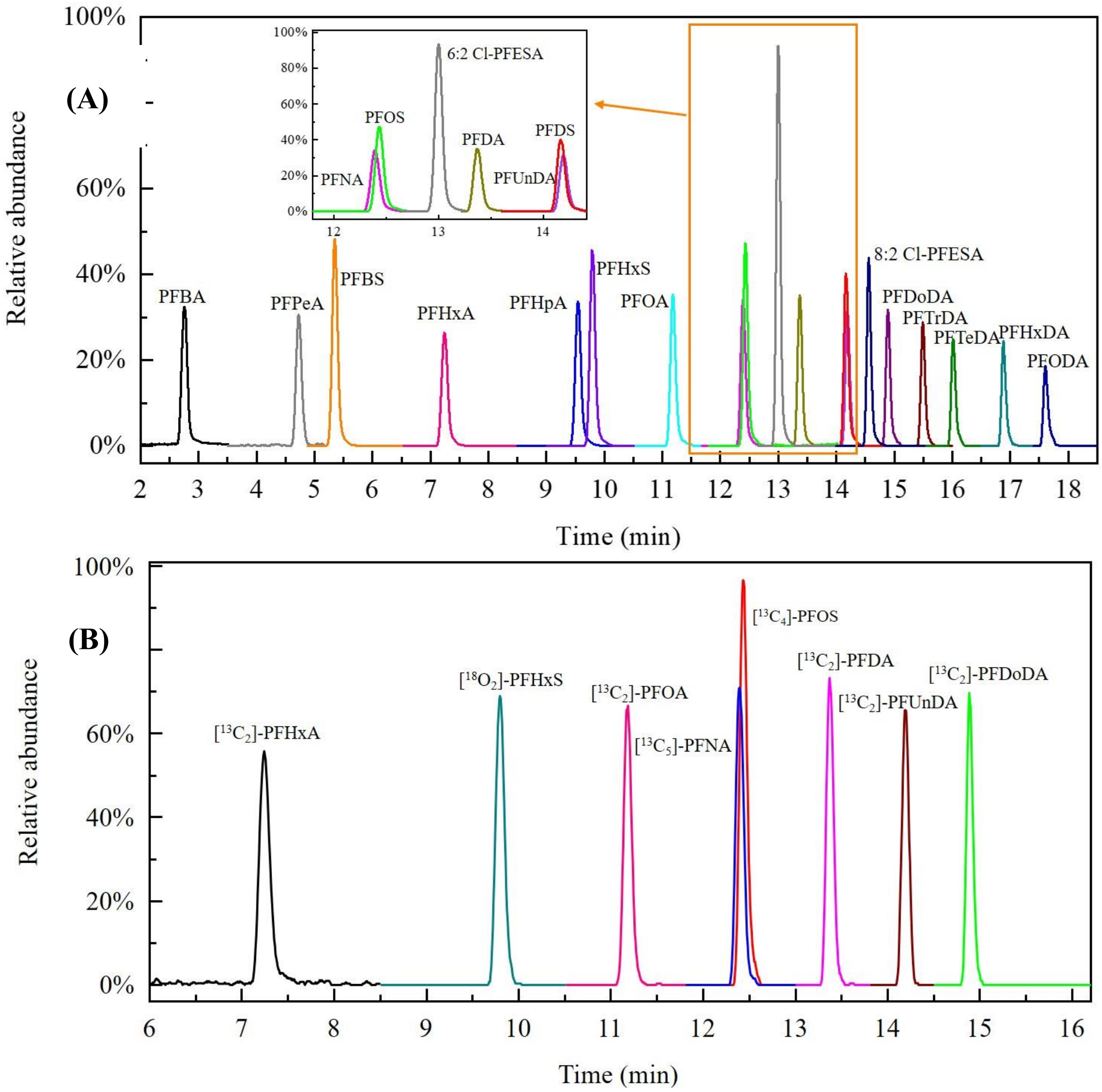

Figure S1. Liquid chromatography coupled to hybrid quadrupole-Orbitrap mass spectrometer LC-MS (Q-Exactive) (Thermos fisher scientific, USA) chromatograms for (A) standard PFASs and their (B) internal standard.

References:

1. Ding, G.; Wouterse, M.; Baerselman, R.; Peijnenburg, W.J.G.M. Toxicity of Polyfluorinated and Perfluorinated Compounds to Lettuce (Lactuca sativa) and Green Algae (Pseudokirchneriella subcapitata). *Archives of Environmental Contamination and Toxicology* **2012**, *62*, 49-55, doi:10.1007/s00244-011-9684-9.
2. Rosal, R.; Rodea-Palomares, I.; Boltes, K.; Fernández-Piñas, F.; Leganés, F.; Petre, A. Ecotoxicological assessment of surfactants in the aquatic environment: Combined toxicity of docusate sodium with chlorinated pollutants. *Chemosphere* **2010**, *81*, 288-293, doi:https://doi.org/10.1016/j.chemosphere.2010.05.050.
3. Xu, D.; Li, C.; Chen, H.; Shao, B. Cellular response of freshwater green algae to perfluorooctanoic acid toxicity. *Ecotoxicology and Environmental Safety* **2013**, *88*, 103-107, doi:https://doi.org/10.1016/j.ecoenv.2012.10.027.
4. USEPA. Comparison of the Toxicity of Sodium Trifluoroacetate, Difluoroacetic Acid, Sodium Monofluoroacetate and Sodium Fluoride to the Alga. **1995**.
5. Hoke, R.A.; Bouchelle, L.D.; Ferrell, B.D.; Buck, R.C. Comparative acute freshwater hazard assessment and preliminary PNEC development for eight fluorinated acids. *Chemosphere* **2012**, *87*, 725-733, doi:https://doi.org/10.1016/j.chemosphere.2011.12.066.
6. Beach, S.A.; Newsted, J.L.; Coady, K.; Giesy, J.P. Ecotoxicological Evaluation of Perfluorooctanesulfonate (PFOS). In *Reviews of Environmental Contamination and Toxicology: Continuation of Residue Reviews*, Albert, L.A., de Voogt, P., Gerba, C.P., Hutzinger, O., Knaak, J.B., Mayer, F.L., Morgan, D.P., Park, D.L., Tjeerdema, R.S., Whitacre, D.M., et al., Eds.; Springer New York: New York, NY, 2006; pp. 133-174.
7. Latała, A.; Nędzi, M.; Stepnowski, P. Acute toxicity assessment of perfluorinated carboxylic acids towards the Baltic microalgae. *Environ. Toxicol. Pharmacol.* **2009**, *28*, 167-171, doi:https://doi.org/10.1016/j.etap.2009.03.010.
8. Boudreau, T.M.; Sibley, P.K.; Mabury, S.A.; Muir, D.G.C.; Solomon, K.R. Laboratory Evaluation of the Toxicity of Perfluorooctane Sulfonate (PFOS) on Selenastrum capricornutum, Chlorella vulgaris, Lemna gibba, Daphnia magna, and Daphnia pulicaria. *Archives of Environmental Contamination and Toxicology* **2003**, *44*, 0307-0313, doi:10.1007/s00244-002-2102-6.
9. Mitchell, R.J.; Myers, A.L.; Mabury, S.A.; Solomon, K.R.; Sibley, P.K. Toxicity of fluorotelomer carboxylic acids to the algae Pseudokirchneriella subcapitata and Chlorella vulgaris, and the amphipod Hyalella azteca. *Ecotoxicology and Environmental Safety* **2011**, *74*, 2260-2267, doi:https://doi.org/10.1016/j.ecoenv.2011.07.034.
10. Yang, S.; Xu, F.; Wu, F.; Wang, S.; Zheng, B. Development of PFOS and PFOA criteria for the protection of freshwater aquatic life in China. *Science of The Total Environment* **2014**, *470-471*, 677-683, doi:https://doi.org/10.1016/j.scitotenv.2013.09.094.
11. Houde, M.; Douville, M.; Giraudo, M.; Jean, K.; Lépine, M.; Spencer, C.; De Silva, A.O. Endocrine-disruption potential of perfluoroethylcyclohexane sulfonate (PFECHS) in chronically exposed Daphnia magna. *Environmental Pollution* **2016**, *218*, 950-956, doi:https://doi.org/10.1016/j.envpol.2016.08.043.
12. Lu, G.-h.; Liu, J.-c.; Sun, L.-s.; Yuan, L.-j. Toxicity of perfluorononanoic acid and perfluorooctane sulfonate to Daphnia magna. *Water Science and Engineering* **2015**, *8*, 40-48, doi:https://doi.org/10.1016/j.wse.2015.01.001.
13. Boudreau, T.M. Toxicity of perfluorinated organic acids to selected freshwater organisms under laboratory and field conditions. 2002.
14. Phillips, M.M.; Dinglasan-Panlilio, M.J.A.; Mabury, S.A.; Solomon, K.R.; Sibley, P.K. Fluorotelomer Acids are More Toxic than Perfluorinated Acids. *Environmental Science & Technology* **2007**, *41*, 7159-7163, doi:10.1021/es070734c.
15. MacDonald, M.M. Fluorotelomer acid toxicity to aquatic organisms. 2006.

16. Annunziato, K.M.; Jantzen, C.E.; Gronske, M.C.; Cooper, K.R. Subtle morphometric, behavioral and gene expression effects in larval zebrafish exposed to PFHxA, PFHxS and 6:2 FTOH. *Aquatic Toxicology* **2019**, *208*, 126-137, doi:https://doi.org/10.1016/j.aquatox.2019.01.009.
17. Stengel, D.; Wahby, S.; Braunbeck, T. In search of a comprehensible set of endpoints for the routine monitoring of neurotoxicity in vertebrates: sensory perception and nerve transmission in zebrafish (Danio rerio) embryos. *Environmental Science and Pollution Research* **2018**, *25*, 4066-4084, doi:10.1007/s11356-017-0399-y.
18. Zheng, X.-M.; Liu, H.-L.; Shi, W.; Wei, S.; Giesy, J.P.; Yu, H.-X. Effects of perfluorinated compounds on development of zebrafish embryos. *Environmental Science and Pollution Research* **2012**, *19*, 2498-2505, doi:10.1007/s11356-012-0977-y.
19. Kalasekar, S.M.; Zacharia, E.; Kessler, N.; Ducharme, N.A.; Gustafsson, J.-Å.; Kakadiaris, I.A.; Bondesson, M. Identification of environmental chemicals that induce yolk malabsorption in zebrafish using automated image segmentation. *Reproductive Toxicology* **2015**, *55*, 20-29, doi:https://doi.org/10.1016/j.reprotox.2014.10.022.
20. Corrales, J.; Kristofco, L.A.; Steele, W.B.; Saari, G.N.; Kostal, J.; Williams, E.S.; Mills, M.; Gallagher, E.P.; Kavanagh, T.J.; Simcox, N.; et al. Toward the Design of Less Hazardous Chemicals: Exploring Comparative Oxidative Stress in Two Common Animal Models. *Chem. Res. Toxicol.* **2017**, *30*, 893-904, doi:10.1021/acs.chemrestox.6b00246.
21. Ulhaq, M.; Carlsson, G.; Örn, S.; Norrgren, L. Comparison of developmental toxicity of seven perfluoroalkyl acids to zebrafish embryos. *Environ. Toxicol. Pharmacol.* **2013**, *36*, 423-426, doi:https://doi.org/10.1016/j.etap.2013.05.004.
22. Huang, H.; Huang, C.; Wang, L.; Ye, X.; Bai, C.; Simonich, M.T.; Tanguay, R.L.; Dong, Q. Toxicity, uptake kinetics and behavior assessment in zebrafish embryos following exposure to perfluorooctanesulphonicacid (PFOS). *Aquatic Toxicology* **2010**, *98*, 139-147, doi:https://doi.org/10.1016/j.aquatox.2010.02.003.
23. Stengel, D.; Zindler, F.; Braunbeck, T. An optimized method to assess ototoxic effects in the lateral line of zebrafish (Danio rerio) embryos. *Comparative Biochemistry and Physiology Part C: Toxicology & Pharmacology* **2017**, *193*, 18-29, doi:https://doi.org/10.1016/j.cbpc.2016.11.001.
24. Li, Y.; Han, Z.; Zheng, X.; Ma, Z.; Liu, H.; Giesy, J.P.; Xie, Y.; Yu, H. Comparison of waterborne and in ovo nanoinjection exposures to assess effects of PFOS on zebrafish embryos. *Environmental Science and Pollution Research* **2015**, *22*, 2303-2310, doi:10.1007/s11356-014-3527-y.
25. Wang, S.; Zhuang, C.; Du, J.; Wu, C.; You, H. The presence of MWCNTs reduces developmental toxicity of PFOS in early life stage of zebrafish. *Environmental Pollution* **2017**, *222*, 201-209, doi:https://doi.org/10.1016/j.envpol.2016.12.055.
26. Ding, G.; Zhang, J.; Chen, Y.; Wang, L.; Wang, M.; Xiong, D.; Sun, Y. Combined Effects of PFOS and PFOA on Zebrafish (Danio rerio) Embryos. *Archives of Environmental Contamination and Toxicology* **2013**, *64*, 668-675, doi:10.1007/s00244-012-9864-2.
27. Hagenaars, A.; Stinckens, E.; Vergauwen, L.; Bervoets, L.; Knapen, D. PFOS affects posterior swim bladder chamber inflation and swimming performance of zebrafish larvae. *Aquatic Toxicology* **2014**, *157*, 225-235, doi:https://doi.org/10.1016/j.aquatox.2014.10.017.
28. Hagenaars, A.; Vergauwen, L.; De Coen, W.; Knapen, D. Structure–activity relationship assessment of four perfluorinated chemicals using a prolonged zebrafish early life stage test. *Chemosphere* **2011**, *82*, 764-772, doi:https://doi.org/10.1016/j.chemosphere.2010.10.076.
29. Shi, G.; Cui, Q.; Pan, Y.; Sheng, N.; Sun, S.; Guo, Y.; Dai, J. 6:2 Chlorinated polyfluorinated ether sulfonate, a PFOS alternative, induces embryotoxicity and disrupts cardiac development in zebrafish embryos. *Aquatic Toxicology* **2017**, *185*, 67-75, doi:https://doi.org/10.1016/j.aquatox.2017.02.002.
30. Cao, Y.; Zhang, Y.; Lei, C.; Liu, Z. Environmental Pollution and Ecological Toxicity of Perfluorinated Chemicals: A Riview. *Journal of Environment and Health* **2012**, *29*, 561-567.
31. Australian Department of Health and Ageing (DoHA). Potassium Perfluorobutane Sulfonate, Existing Chemical Hazard Assessment. **2005**.

32. Wang, X.; Fan, B.; Fan, M.; Belanger, S.; Li, J.; Chen, J.; Gao, X.; Liu, Z. Development and use of interspecies correlation estimation models in China for potential application in water quality criteria. *Chemosphere* **2020**, *240*, doi:10.1016/j.chemosphere.2019.124848.
33. Rodea-Palomares, I.; Makowski, M.; Gonzalo, S.; González-Pleiter, M.; Leganés, F.; Fernández-Piñas, F. Effect of PFOA/PFOS pre-exposure on the toxicity of the herbicides 2,4-D, Atrazine, Diuron and Paraquat to a model aquatic photosynthetic microorganism. *Chemosphere* **2015**, *139*, 65-72, doi:https://doi.org/10.1016/j.chemosphere.2015.05.078.
34. Rodea-Palomares, I.; Leganés, F.; Rosal, R.; Fernández-Piñas, F. Toxicological interactions of perfluorooctane sulfonic acid (PFOS) and perfluorooctanoic acid (PFOA) with selected pollutants. *Journal of Hazardous Materials* **2012**, *201-202*, 209-218, doi:https://doi.org/10.1016/j.jhazmat.2011.11.061.
35. Hu, C.; Luo, Q.; Huang, Q. Ecotoxicological effects of perfluorooctanoic acid on freshwater microalgae Chlamydomonas reinhardtii and Scenedesmus obliquus. *Environmental Toxicology and Chemistry* **2014**, *33*, 1129-1134, doi:https://doi.org/10.1002/etc.2532.
36. Zhang, L.; Niu, J.; Li, Y.; Wang, Y.; Sun, D. Evaluating the sub-lethal toxicity of PFOS and PFOA using rotifer Brachionus calyciflorus. *Environmental Pollution* **2013**, *180*, 34-40, doi:https://doi.org/10.1016/j.envpol.2013.04.031.
37. Yuan, Z.; Zhang, J.; Zhang, Y.; Zhen, H.; Sun, Y. The Effect of Perfluorooctanoic Acid on the Planarian <i>Dugesia japonica</i>. *Polish Journal of Environmental Studies* **2015**, *24*, 801-807, doi:10.15244/pjoes/32098.
38. Mhadhbi, L.; Rial, D.; Pérez, S.; Beiras, R. Ecological risk assessment of perfluorooctanoic acid (PFOA) and perfluorooctanesulfonic acid (PFOS) in marine environment using Isochrysis galbana, Paracentrotus lividus, Siriella armata and Psetta maxima. *Journal of Environmental Monitoring* **2012**, *14*, 1375-1382, doi:10.1039/C2EM30037K.
39. Li, M.-H. Effects of nonionic and ionic surfactants on survival, oxidative stress, and cholinesterase activity of planarian. *Chemosphere* **2008**, *70*, 1796-1803, doi:https://doi.org/10.1016/j.chemosphere.2007.08.032.
40. Hazelton, P.D.; Cope, W.G.; Pandolfo, T.J.; Mosher, S.; Strynar, M.J.; Barnhart, M.C.; Bringolf, R.B. Partial life-cycle and acute toxicity of perfluoroalkyl acids to freshwater mussels. *Environmental Toxicology and Chemistry* **2012**, *31*, 1611-1620, doi:https://doi.org/10.1002/etc.1866.
41. Su, H.; Ren, D.; Cao, S.; Qin, Z. Acute Toxicity of Perfluorooctane Sulfonate (PFOS) and Its Substitutes to Amphibian Tadpoles. *Asian Journal of Ecotoxicology* **2012**, *7*, 521-524.
42. Wei, R.; Su, H.; Qin, Z. Developmental Toxicity of Perfluorooctane Sulfonate, Perfluorohexane Sulfonate and Perfluorobutane Sulfonate to Xenopus Embryos. *Asian Journal of Ecotoxicology* **2012**, *7*, 542-548.
43. Wang, H.; Ma, S.; Zhang, Z.; Chen, H.; Huang, Z.; Gong, X.; Cai, W.; Jia, X. Effects of Perfluorooctane Sulfonate (PFOS) Exposure on Antioxidant Enzymes of Perna viridis. *Asian Journal of Ecotoxicology* **2012**, *7*, 508-516.
44. Wang, H. Effects of Perfluorooctanesulfonate on Antioxidant System, Tissues Injury and Genes Expression of Perna viridis and Pagrosomus major. Shanghai Ocean University, Shanghai, China, 2012.
